# Dietary Sodium and Potassium Intake and Risk of Non-Fatal Cardiovascular Diseases: The Million Veteran Program

**DOI:** 10.3390/nu14051121

**Published:** 2022-03-07

**Authors:** Dong D. Wang, Yanping Li, Xuan-Mai T. Nguyen, Rebecca J. Song, Yuk-Lam Ho, Frank B. Hu, Walter C. Willett, Peter W. F. Wilson, Kelly Cho, J. Michael Gaziano, Luc Djoussé, on behalf of the VA Million Veteran Program

**Affiliations:** 1 Massachusetts Veterans Epidemiology Research and Information Center (MAVERIC), VA Boston Healthcare System, Boston, MA 02111, USA; yanping.li@va.gov (Y.L.); xuan-mai.nguyen@va.gov (X.-M.T.N.); rebecca.song@va.gov (R.J.S.); yuk-lam.ho@va.gov (Y.-L.H.); kelly.cho@va.gov (K.C.); michael.gaziano@va.gov (J.M.G.); ldjousse@rics.bwh.harvard.edu (L.D.); 2 The Channing Division of Network Medicine, Department of Medicine, Brigham and Women’s Hospital, Harvard Medical School, Boston, MA 02115, USA; fhu@hsph.harvard.edu (F.B.H.); wwillett@hsph.harvard.edu (W.C.W.); 3 Department of Nutrition, Harvard T. H. Chan School of Public Health, Boston, MA 02115, USA; 4 Division of Aging, Department of Medicine, Brigham and Women′s Hospital, Boston, MA 02115, USA; 5 Harvard Medical School, Boston, MA 02115, USA; 6 Department of Epidemiology, Boston University School of Public Health, Boston, MA 02115, USA; 7 Department of Epidemiology, Harvard T. H. Chan School of Public Health, Boston, MA 02115, USA; 8 Atlanta VA Medical Center, Atlanta, GA 30033, USA; peter.wilson@va.gov; 9 Emory Clinical Cardiovascular Research Institute, Atlanta, GA 30033, USA

**Keywords:** cardiovascular disease, sodium, potassium, veterans, nutrition, epidemiology

## Abstract

Objective: To examine the association between intakes of sodium and potassium and the ratio of sodium to potassium and incident myocardial infarction and stroke. Design, Setting and Participants: Prospective cohort study of 180,156 Veterans aged 19 to 107 years with plausible dietary intake measured by food frequency questionnaire (FFQ) who were free of cardiovascular disease (CVD) and cancer at baseline in the VA Million Veteran Program (MVP). Main outcome measures: CVD defined as non-fatal myocardial infarction (MI) or acute ischemic stroke (AIS) ascertained using high-throughput phenotyping algorithms applied to electronic health records. Results: During up to 8 years of follow-up, we documented 4090 CVD cases (2499 MI and 1712 AIS). After adjustment for confounding factors, a higher sodium intake was associated with a higher risk of CVD, whereas potassium intake was inversely associated with the risk of CVD [hazard ratio (HR) comparing extreme quintiles, 95% confidence interval (CI): 1.09 (95% CI: 0.99–1.21, *p* trend = 0.01) for sodium and 0.87 (95% CI: 0.79–0.96, *p* trend = 0.005) for potassium]. In addition, the ratio of sodium to potassium (Na/K ratio) was positively associated with the risk of CVD (HR comparing extreme quintiles = 1.26, 95% CI: 1.14–1.39, *p* trend < 0.0001). The associations of Na/K ratio were consistent for two subtypes of CVD; one standard deviation increment in the ratio was associated with HRs (95% CI) of 1.12 (1.06–1.19) for MI and 1.11 (1.03–1.19) for AIS. In secondary analyses, the observed associations were consistent across race and status for diabetes, hypertension, and high cholesterol at baseline. Associations appeared to be more pronounced among participants with poor dietary quality. Conclusions: A high sodium intake and a low potassium intake were associated with a higher risk of CVD in this large population of US veterans.

## 1. Introduction

High sodium intake has been shown to be associated with a higher risk of hypertension and cardiovascular morbidity and mortality in an approximately linear dose-response manner [1,2,3]. For example, a recent meta-analysis of 261,732 participants with 10,150 cases of stroke reported that dietary sodium intake and sodium-to-potassium ratio were positively associated with risk of stroke without evidence of nonlinearity [4]. Based on this dose-response relationship, modeling-based studies estimated that 1.65 million cardiovascular deaths globally [1] and more than 50,000 myocardial infarctions and 30,000 strokes per year in the US were attributable to high sodium consumption, defined as intake level above 2.0 g per day [2]. However, several recent studies have challenged the aforementioned evidence and reported a J-shaped dose-response relationship with the lowest risk of death and cardiovascular events observed at a urinary sodium excretion level of 3 to 6 g/day [5]. 

This inconsistency of the evidence may be partially explained by measurement errors embedded in the ascertainment of urinary sodium level, especially in those studies that relied on 24-h urinary sodium level imputed from spot urine samples [6,7]. In addition, 24-h urinary sodium excretion can only reflect short-term intake level, whereas long-term usual intakes of sodium and potassium are biologically more relevant for CVD with a long induction period. To address these gaps, we examined the associations of long-term intakes of sodium and potassium, measured by a semi-quantitative food frequency questionnaire (sFFQ), with incident myocardial infarction and stroke in a large cohort of US veterans with diverse socioeconomic and racial/ethnic backgrounds.

## 2. Methods

### 2.1. Study Population

Million Veteran Program (MVP) is a nationally representative, prospective cohort study of veterans designed to study genetic and nongenetic determinants of chronic diseases. Recruitment and enrollment for MVP began in early 2011 among eligible Veterans receiving routine primary care within the US Department of Veterans Affairs Healthcare System. Participant data is collected through self-reported surveys and electronic health records (EHR). Details of the study design can be found elsewhere [8]. All participants gave informed consent, and the Veterans Affairs Central Institutional Review Board approved the study protocol.

**Figure 1:**
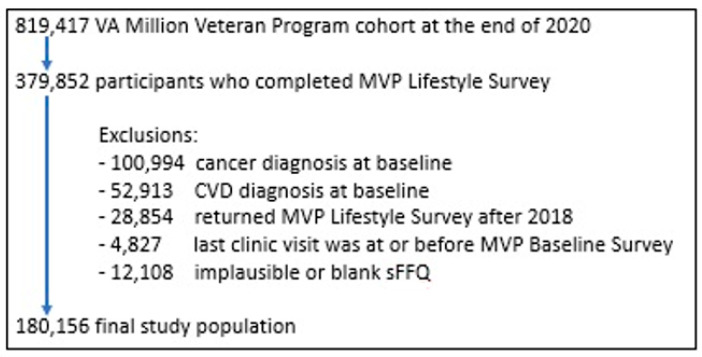
Participant Flow Chart.

As of 2020, MVP consisted of 819,417 enrollees of which 379,852 participants completed the MVP Lifestyle Survey. For the current analysis, we excluded participants with a self-reported history of cancer (*n* = 100,994) or CVD (*n* = 52,913) at baseline where CVD history included self-reported diagnosis of stroke, coronary artery/coronary heart disease (includes angina) or confirmed MI or AIS. Participants who responded to the MVP Lifestyle Survey after December 2018 (*n* = 28,854) and whose last clinical visit was before or on the date of MVP Baseline Survey completion (*n* = 4827) were also excluded. The MVP Lifestyle Survey includes a sFFQ with a total of 61 food items. Those who did not provide dietary information or reported implausible dietary intakes at baseline (*n* = 12,108) were not included in this study, yielding a final population of 180,156 participants (Figure 1).

### 2.2. Assessment of Exposure and Covariates

Dietary intake was self-reported through a sFFQ, which has been previously validated in other cohorts [9]. Participants were asked “how often” they consume a standard portion of each food item in the past year. Pre-specified responses were: “Never or less than once per month”; “1–3 per month”; “once a week”; “2–4 per week”; “5–6 per week”; “once a day”; “2–3 per day”; “4–5 per day”; and “≥6 per day”. Frequencies and portions of each food item were converted to average daily intake for each participant. Nutrient intake and total energy intake were calculated by multiplying the frequency of consumption for each food item by its energy and nutrient content from the Harvard University Food Composition Database [10] and summing across all foods. Nutrients were adjusted for total energy intake using a residual method [11]. Baseline data on age, sex, race, family income, education, body mass index, alcohol consumption, exercise, smoking, dietary supplements, comorbidities, and medications were taken from the self-reported MVP Baseline Survey.

### 2.3. Assessment of Cardiovascular Diseases

Using combined information from the VA Corporate Data Warehouse (CDW) which is the VA EHR, Centers for Medicaid & Medicare Services database, and National Death Index database [12,13], we focused on cases of non-fatal myocardial infarction (MI) and acute ischemic stroke (AIS) [14,15,16]. MI and AIS cases were identified by applying the Surrogate-Assisted Feature Extraction (SAFE) method, a validated high-throughput phenotyping algorithms approach, using a combination of (International Classification of Diseases) ICD codes, natural language processing, and medical record review labels [17,18].

### 2.4. Statistical Analysis

Person-years of follow-up were calculated from sFFQ assessment to the first occurrence of MI or AIS, death, the end of follow-up (31 December 2018), or last visit recorded in CDW. We used Cox proportional hazards models to estimate hazard ratios (HRs) with 95% confidence intervals (CIs), comparing higher quintiles to the lowest quintile of dietary indices with simultaneous adjustment for covariates. Covariates included age (continuous), sex (male or female), race (white or other), education level (≤high school or GED (General Educational Diploma), some colleague, or college or above), income level (<$30,000, $30,000–$59,000 or ≥$60,000), marital status (currently married or not), smoking status (current, former or never smoking), frequency of alcohol consumption (never, <1 time/week or ≥1 time/week), frequency of vigorous exercise (never/rarely, 1–4 times/month, 2–4 times/week, or ≥5 times/week), dietary energy intake (quintiles), body mass index (continuous), and the presence of diabetes, hypertension or high cholesterol at baseline. 

To quantify a linear trend of relative risk of CVD across quintiles, we assigned the median within each quintile and modeled this variable continuously; the Wald test was used to assess statistical significance. We also tested for potential nonlinearity in the association between dietary indices and the risk of CVD. Restricted cubic spline regression with 3 knots was applied to flexibly model the association between the dietary indices and risk of CVD with the first percentile of each dietary indices as the reference. Nonlinearity in the dose-response relationship of the dietary indices with the risk of CVD was evaluated by comparing the model with the linear term to the model with the linear and cubic spline terms using the likelihood ratio test [19]. 

In secondary analyses, we examined the association between sodium to potassium ratio and the risk of CVD among White and Black participants separately. We also conducted stratified analyses to examine the association between the ratio of sodium to potassium and the risk of CVD across different participant subgroups: with and without baseline diabetes, hypertension, or high cholesterol and by overall dietary quality. Overall dietary quality was evaluated by Dietary Approaches to Stop Hypertension (DASH) score [20]. We tested for interactions between the ratio of sodium to potassium and the stratification variables by adding a product term of the two variables and their main effects in the multivariable model. 

In sensitivity analyses, we further adjusted for the number of medical diagnoses (0–2, 2–4, 5–6, ≥7) and medications (0, 1, 2, ≥3) at baseline for chronic health conditions in addition to the number of dietary supplements that were taken regularly to test the robustness of findings when we considered multi-medication, multi-morbidity, and dietary supplementation uses in our study population [21]. All analyses were completed using SAS Enterprise Guide 8.2.

## 3. Results

The average energy intake was 1372 kcal/day for women and 1447 kcal/day for men. The observed energy-adjusted average intakes of sodium and potassium were 1246 mg/day (1112 for women; 1262 for men) and 2600 mg/day (2534 for women and 2608 for men), respectively, with an average sodium to potassium ratio (Na/K ratio) of 0.5 for both women and men. Table 1 presents baseline characteristics of participants according to quintiles of sodium, potassium, and Na/K ratio. Participants with higher sodium intake and Na/K ratio were more likely to be inactive, have lower educational attainment, lower annual family income, more chronic conditions, and poor dietary quality.

**Figure 2:**
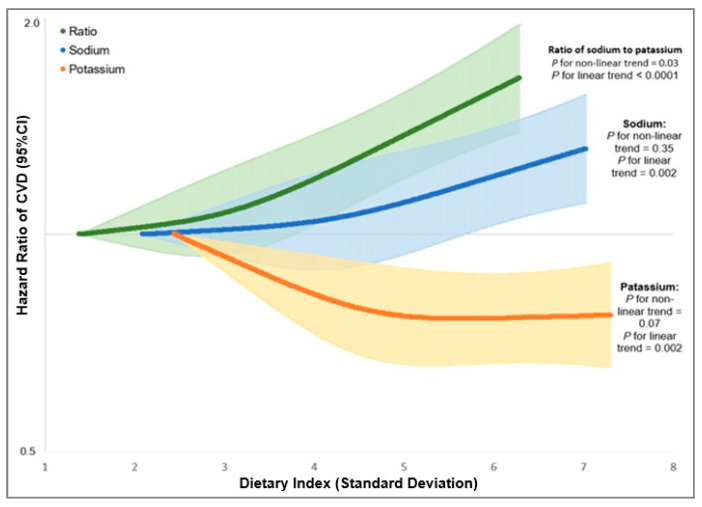
Dose-response relationship between dietary intakes of sodium, potassium, and ratio of sodium to potassium with risk of non-fatal cardiovascular diseases in the Million Veteran Program (The dose-response relationship was quantified by Cox proportional hazards models with restricted cubic spline with 3 knots specified. The first percentile of each dietary indices was used as reference level for calculating hazard ratios. We tested nonlinearity in the dose-response relationship of the dietary indices with CVD risk by comparing the model with only the linear term to the model with the linear and the cubic spline terms and using the likelihood ratio test. All the models simultaneously adjusted for age (continuous), sex (male or female), race/ethnicity, education level, income, and marital status, smoking status, frequency of alcohol consumption, frequency of exercise vigorously, total energy intake (in quintiles), body mass index (continuous), and the presences of diabetes, high cholesterol and hypertension at baseline (each Yes vs. No).).

During a mean follow-up of 3.5 years (up to 8 years), we documented 4090 CVD events, including 2499 MI and 1712 AIS cases (121 participants developed both MI and AIS). After adjusting for known and suspected confounding variables and risk factors, a higher sodium intake was associated with a higher risk of CVD (HR comparing extreme quintiles = 1.09, 95% CI: 0.99–1.21, *p* trend = 0.01). Cubic spline model revealed an approximately linear dose-response association between sodium intake and CVD risk without any indication of nonlinearity (*p* for non-linear = 0.35 and *p* for linear = 0.002). Across quintiles, a higher potassium intake was associated with a 13% lower risk of total CVD (HR comparing extreme quintiles = 0.87, 95% CI: 0.79–0.96, *p* trend = 0.005). The dose-response association between potassium and risk of CVD (*p* for non-linear = 0.07 and *p* for linear = 0.002) was slightly nonlinear; the decreasing trend plateaued after about 3 g/day. We found that Na/K ratio was positively associated with the risk of total CVD (HR comparing extreme quintiles = 1.26, 95% CI: 1.14–1.39, *p* trend < 0.0001). Similar to the association of sodium intake and CVD risk, the association between Na/K ratio and risk of CVD was linear (*p* for non-linear = 0.15 and *p* for linear < 0.0001, Figure 2). When modeling intake level continuously, every 1-SD increment in intake level was associated with a 6% higher risk of CVD (HR = 1.06, 95% CI: 1.01–1.10) for sodium, a 5% lower risk of CVD (HR = 0.95, 95% CI: 0.91–0.98) for potassium, and an 11% higher risk of CVD for Na/K ratio (HR = 1.11, 95% CI: 1.06–1.16) (Table 2). The associations did not materially change after further adjustment for baseline health conditions, medication usage, and dietary supplements (every 1-SD increment in sodium HR = 1.05, 95% CI: 1.01–1.10; potassium HR = 0.95, 95% CI: 0.92–0.99; Na/K ratio HR = 1.10, 95% CI: 1.05–1.16). 

We found similar associations between Na/K ratio and subtypes of CVD. Every 1-SD increment in Na/K ratio was associated with HRs (95% CI) of 1.12 (1.06–1.19) for non-fatal MI and 1.11 (1.03–1.19) for non-fatal AIS (Table 3). The association between Na/K ratio and CVD risk was consistent between White and Black participants and between participants with or without diabetes, hypertension, or high cholesterol at baseline whereas the association appeared to be slightly pronounced in participants with poor dietary quality (all *P*s for interaction > 0.05, Table 4).

## 4. Discussion

In this large cohort of US veterans, we observed that higher dietary sodium intake and Na/K ratio were each significantly associated with a higher risk of CVD in a linear dose-response manner. In addition, we observed a nonlinear inverse association between dietary potassium intake and risk of CVD; the lowest risk was observed for 3 g/day. The observed associations were consistent across racial groups and participants with or without baseline cardiometabolic conditions but appeared to be slightly stronger among veterans with a low dietary quality. We found similar positive associations of the Na/K ratio with both MI and AIS. 

Our findings are consistent with previous reports assessing the relationship between sodium intake and CVD [22,23]. For example, Cook et al. [22] reported a linear association of sodium with cardiovascular events where every 1000 mg/d increase in sodium was associated with a 17% higher risk (*p* = 0.05). In addition, high blood pressure has been positively associated with CVD risk [24] and the DASH (Dietary Approaches to Stop Hypertension)-Sodium clinical trial demonstrated beneficial effects of sodium reduction on blood pressure, even at very low levels of sodium intake (1500 mg/d). [25]. However, several studies challenged this evidence and proposed a J-shaped dose-response relationship with the lowest risk of death and cardiovascular events observed at a urinary sodium excretion level of 3 to 6 g/day. Potential limitations of these studies include measurement errors in sodium excretion, reverse causation, and residual confounding [3]. In contrast, our data do not support any potential risk associated with very low sodium intake (1500 to 2300 mg/d). In addition, a systematic review by the Institute of Medicine concluded that the underlying data supporting the potential high risk of CVD associated with very low sodium intake were of insufficient quality [26]. 

High sodium and low potassium intakes have been associated with increased CVD risk through potential elevated blood pressure effects [1,2,3,4,5]. In a meta-analysis of randomized clinical trials, reduced sodium intake, increased potassium intake, and use of potassium-containing salt substitutes in the diet reduced blood pressure [27]. A recent meta-analysis of prospective and retrospective observational studies reported that a higher sodium intake and higher sodium-to-potassium ratio were associated with a higher risk of stroke [5]. Our observed positive association between the ratio of sodium to potassium and CVD is consistent with previous studies based on FFQ, dietary records, 24 h urine excretion, and biomarker-calibrated intakes [28,29,30,31]. 

The current study has several strengths including a prospective cohort design with a large sample size of participants with diverse socioeconomic and racial/ethnic backgrounds, a large number of confirmed CVD cases, and comprehensive measurements and careful adjustment for many potential confounders, and complete ascertainment of CVD events via EHR. Our study has several limitations. First, the abbreviated FFQ in MVP likely underestimates the dietary sodium and potassium intakes. Some major sources of dietary sodium were missed in the MVP FFQ, such as salt added at the table and salt used in preparation or cooking [32,33]. Therefore, it is hard to distinguish whether the findings reflect the effects of sodium intake per se or a dietary pattern high in sodium. Second, our single assessment of dietary intake precluded the assessment of changes in sodium and potassium intakes over time. In addition, our use of a self-reported questionnaire as the primary means to collect dietary data is a limitation; measurement errors in dietary assessment are inevitable. However, we were able to control for measurement error to a large degree by applying energy adjustment that negated correlated errors in nutrient and energy intake assessments [11]. Third, the prevalence of major chronic diseases was high in the MVP population at baseline. Participants with concerns about an illness might have changed their dietary behaviors, potentially leading to biased results. However, to minimize this bias, we excluded participants with major chronic disease at baseline and performed statistical adjustment for diabetes, hypertension, and dyslipidemia. Lastly, hemorrhagic stroke was not included in our CVD identification because phenotyping efforts for this outcome are still ongoing in MVP; we expect to examine sodium and potassium intake in relation to hemorrhagic stroke in a future project.

In conclusion, our data showed that while sodium intake is positively associated with CVD risk, the reverse is true for potassium intake among US veterans. These findings support the recommendation of dietary patterns with lower dietary sodium to potassium ratio to prevent major cardiovascular diseases. Furthermore, our findings support the Dietary Guideline for Americans [34] and the World Health Organization guideline of reducing sodium intake and increasing potassium intake to reduce the risk of CVD, stroke, and coronary heart disease in adults [35,36]. 

## Figures and Tables

**Table 1:** Baseline characteristics of 180,156 participants in the Million Veteran Program according to energy-adjusted dietary intakes of sodium and potassium and ratio of sodium to potassium.

	Sodium			Potassium			Ratio of Sodium to Potassium		
	Q1	Q3	Q5	Q1	Q3	Q5	Q1	Q3	Q5
***N***	36,031	36,032	36,031	36,031	36,032	36,031	36,031	36,032	36,031
**Sodium, mg/d**	865	1231	1666	1181	1271	1251	973	1242	1532
**Potassium, mg/d**	2478	2634	2628	1857	2575	3406	3102	2596	2112
**Ratio of sodium to potassium**	0.4	0.5	0.7	0.6	0.5	0.4	0.3	0.5	0.7
**Age, years**	59.9	62.3	64.2	59.3	62.6	64.2	62.2	62.4	61.9
**Men, %**	80.8	90.5	95.7	87.0	89.6	90.8	84.0	89.9	92.8
**White, %**	75.4	83.0	86.7	75.1	83.5	85.4	79.3	83.5	80.9
**Black, %**	18.3	13.1	10.1	20.0	12.5	10.3	14.9	12.4	15.6
**Married, %**	51.9	60.2	59.5	49.1	61.0	60.9	56.3	61.0	54.1
**Education level, %**									
**≤High school or GED**	19.3	21.4	28.1	27.4	20.9	21.0	17.6	21.6	29.2
**Some colleague**	29.3	30.1	31.9	32.6	29.4	29.2	28.0	30.4	32.6
**College or above**	51.4	48.5	39.9	40.0	49.7	49.8	54.4	48.0	38.3
**Annual family income, %**									
**<30,000**	32.9	31.9	39.1	40.8	31.5	32.8	31.4	31.5	41.2
**30,000–59,000**	33.8	35.6	35.9	33.7	35.5	35.5	34.0	35.9	34.5
**≥60,000**	33.3	32.4	24.9	25.6	33.0	31.7	34.6	32.6	24.2
**Smoking status, %**									
**Never smoking**	35.7	33.4	27.8	30.3	33.3	32.6	36.6	32.5	29.1
**Former smoker**	56.7	59.1	64.5	59.7	59.5	61.3	57.4	60.0	62.0
**Current smoking**	7.6	7.5	7.7	10.0	7.2	6.1	6.0	7.5	8.9
**Vigorously exercise ^1^, %**									
**Never/rarely**	27.1	26.5	33.7	37.4	26.2	24.2	22.6	27.6	36.9
**1–4 times/month**	24.5	27.3	27.2	27.8	27.8	23.9	23.0	27.7	27.5
**2–4 times/week**	31.9	31.9	26.5	23.7	31.9	33.5	35.0	31.0	24.1
**≥5 times/week**	16.5	14.3	12.7	11.1	14.1	18.4	19.4	13.7	11.6
**Alcohol drinking, %**									
**Never**	34.0	39.1	50.7	37.5	39.2	48.1	40.2	39.1	46.5
**<1 times/week**	23.7	29.6	30.3	24.3	28.5	30.5	27.1	28.6	28.2
**≥1 times/week**	42.3	31.3	19.0	38.2	32.3	21.4	32.7	32.3	25.3
**Energy intake, kcal/day**	1432	1438	1441	1431	1457	1419	1410	1459	1425
**BMI, kg/m^2^**	28.6	29.6	30.6	29.4	29.7	29.6	28.8	29.7	30.3
**Hypertension, %**	48.5	54.4	62.1	54.2	54.4	55.2	49.5	55.4	58.9
**High cholesterol, %**	45.4	50.0	54.9	47.0	50.2	51.9	47.5	50.9	51.4
**Diabetes, %**	12.9	20.7	35.6	17.3	23.2	26.2	17.2	22.8	27.7
**DASH score**	21.7	21.8	20.7	16.9	21.9	25.5	24.9	21.7	18.0
**Number of baseline diseases and conditions**									
**0–2**	32.1	29.9	28.1	30.6	29.3	29.5	31.7	29.2	29.2
**3–4**	24.8	26.3	25.6	25.1	26.0	25.4	25.1	25.8	25.3
**5–6**	18.4	19.1	19.8	18.8	19.3	19.2	18.5	19.6	19.5
**≥7**	24.7	24.7	26.5	25.4	25.3	25.9	24.8	25.5	26.0
**Number of medication types**									
**0**	31.4	29.6	27.3	28.9	29.1	29.8	31.6	28.5	27.8
**1**	22.0	22.7	23.2	22.4	22.7	22.3	22.2	22.9	22.9
**2**	15.5	16.7	16.7	16.0	16.8	16.0	15.4	16.5	16.5
**≥3**	31.0	30.9	32.8	32.7	31.4	32.0	30.7	32.1	32.7
**Number of dietary supplement types**									
**0**	24.3	25.3	27.1	31.3	24.7	21.7	20.9	25.3	30.1
**1**	21.1	22.6	24.6	24.6	22.8	21.5	20.2	23.0	25.2
**2–3**	29.0	29.4	29.1	26.9	29.8	29.8	29.9	29.8	27.7
**≥4**	25.5	22.7	19.2	17.2	22.7	27.0	28.9	21.9	17.0

Unless otherwise indicated, data are expressed as means; DASH: Dietary Approaches to Stop Hypertension; ^1^ Exercise vigorously enough to work up a sweat.

**Table 2:** Hazard ratios (95% CI) for cardiovascular disease by quintiles (or per SD) of dietary sodium, potassium, and their ratio in 180,156 participants from the Million Veteran Program (2011 to 2018).

	Quintiles of Dietary Indices					*p*_trend_	HR (95%CI)
	Q1	Q2	Q3	Q4	Q5		Per 1-SD Increment
**Sodium Intakes**							
Median	898	1095	1231	1373	1605		
Cases	679	705	766	888	1052		
IR (/10^3^ PYs)	5.47	5.66	6.16	7.12	8.45		
Model 1	1.0 (ref.)	0.98 (0.88, 1.09)	1.02 (0.92, 1.14)	1.15 (1.04, 1.27)	1.33 (1.21, 1.46)	<0.0001	1.16 (1.11, 1.21)
Model 2	1.0 (ref.)	0.97 (0.88, 1.08)	1.00 (0.90, 1.11)	1.10 (0.99, 1.21)	1.19 (1.07, 1.31)	<0.0001	1.10 (1.05, 1.14)
Model 3	1.0 (ref.)	0.97 (0.87, 1.08)	0.99 (0.90, 1.10)	1.08 (0.98, 1.20)	1.17 (1.05, 1.29)	0.0001	1.09 (1.04, 1.13)
Model 4	1.0 (ref.)	0.96 (0.86, 1.06)	0.97 (0.88, 1.08)	1.04 (0.94, 1.16)	1.09 (0.99, 1.21)	0.01	1.06 (1.01, 1.10)
**Potassium Intakes**							
Median	1920	2306	2575	2854	3299		
Cases	830	808	767	833	852		
IR (/10^3^ PYs)	6.85	6.62	6.21	6.60	6.59		
Model 1	1.0 (ref.)	0.90 (0.82, 1.00)	0.81 (0.74, 0.90)	0.84 (0.76, 0.93)	0.83 (0.75, 0.91)	<0.0001	0.93 (0.90, 0.96)
Model 2	1.0 (ref.)	0.97 (0.88, 1.07)	0.89 (0.81, 0.99)	0.93 (0.85, 1.03)	0.90 (0.82, 0.99)	0.03	0.96 (0.93, 1.00)
Model 3	1.0 (ref.)	0.96 (0.87, 1.06)	0.89 (0.80, 0.98)	0.92 (0.84, 1.02)	0.89 (0.81, 0.98)	0.02	0.96 (0.92, 0.99)
Model 4	1.0 (ref.)	0.95 (0.86, 1.05)	0.87 (0.79, 0.96)	0.90 (0.82, 1.00)	0.87 (0.79, 0.96)	0.005	0.95 (0.91, 0.98)
**Ratio of Sodium to Potassium**							
Median	0.33	0.41	0.48	0.55	0.69		
Cases	698	785	756	832	1019		
IR (/10^3^ PYs)	5.48	6.26	6.07	6.75	8.37		
Model 1	1.0 (ref.)	1.12 (1.01, 1.24)	1.09 (0.98, 1.20)	1.21 (1.10, 1.34)	1.52 (1.38, 1.67)	<0.0001	1.23 (1.18, 1.29)
Model 2	1.0 (ref.)	1.12 (1.01, 1.24)	1.04 (0.94, 1.16)	1.12 (1.01, 1.24)	1.31 (1.19, 1.45)	<0.0001	1.14 (1.08, 1.19)
Model 3	1.0 (ref.)	1.12 (1.01, 1.24)	1.04 (0.94, 1.15)	1.11 (1.00, 1.23)	1.30 (1.18, 1.43)	<0.0001	1.13 (1.08, 1.18)
Model 4	1.0 (ref.)	1.10 (0.99, 1.22)	1.02 (0.92, 1.13)	1.08 (0.98, 1.20)	1.26 (1.14, 1.39)	<0.0001	1.11 (1.06, 1.16)

Abbreviation: IR: Incidence rate; PYs: person years. Model 1: age (continuous) and sex (male or female) adjusted relative risk. Model 2 further adjusted for race/ethnicity (European Americans or other), education level (≤high school or GED, some colleague, or college or above), income level (<$30,000, $30,000–$59,000 or ≥$60,000) and marital status (currently married or not), smoking status (current, former or never smoking), frequency of alcohol consumption (never, <1 times/week or ≥1 times/week), frequency of exercise vigorously (never/rarely, 1–4 times/month, 2–4 times/week, or ≥5 times/week). Model 3 further adjusted total energy intake (in quintiles), and body mass index (continuous). Model 4 further adjusted the presences of diabetes, high cholesterol, and hypertension at baseline (each Yes vs. No).

**Table 3:** Hazard ratios (95% CI) for subtypes of cardiovascular disease by quintiles (or per SD) of dietary sodium, potassium, and their ratio in 180,156 participants from the Million Veteran Program (2011 to 2018).

		Quintile of Dietary Indices					*p*_trend_	HR (95%CI)
		Q1	Q2	Q3	Q4	Q5		Per 1-SD Increment
	**Sodium**							
Non-fatal MI	Cases	393	422	489	554	641		
	Model1	1.0 (ref.)	1.01 (0.88, 1.15)	1.12 (0.98, 1.28)	1.23 (1.08, 1.40)	1.38 (1.21, 1.56)	<0.0001	1.17 (1.11, 1.24)
	Model2	1.0 (ref.)	0.99 (0.86, 1.14)	1.07 (0.94, 1.23)	1.13 (1.00, 1.29)	1.18 (1.04, 1.35)	0.001	1.09 (1.04, 1.15)
	Model3	1.0 (ref.)	0.98 (0.86, 1.13)	1.06 (0.93, 1.21)	1.11 (0.98, 1.27)	1.15 (1.01, 1.31)	0.005	1.08 (1.02, 1.14)
	Model4	1.0 (ref.)	0.97 (0.85, 1.12)	1.04 (0.91, 1.19)	1.08 (0.95, 1.23)	1.09 (0.96, 1.24)	0.06	1.05 (1.00, 1.11)
Non-fatal AIS	Cases	306	300	297	363	446		
	Model1	1.0 (ref.)	0.93 (0.79, 1.09)	0.89 (0.76, 1.05)	1.06 (0.91, 1.23)	1.27 (1.1, 1.47)	<0.0001	1.14 (1.07, 1.22)
	Model2	1.0 (ref.)	0.94 (0.8, 1.11)	0.9 (0.77, 1.06)	1.05 (0.9, 1.23)	1.2 (1.04, 1.4)	0.0018	1.11 (1.04, 1.18)
	Model3	1.0 (ref.)	0.94 (0.8, 1.11)	0.9 (0.77, 1.06)	1.05 (0.9, 1.22)	1.2 (1.03, 1.39)	0.0026	1.11 (1.04, 1.18)
	Model4	1.0 (ref.)	0.93 (0.79, 1.09)	0.88 (0.75, 1.03)	1 (0.85, 1.17)	1.1 (0.94, 1.28)	0.0754	1.06 (0.99, 1.13)
	**Potassium**							
Non-Fatal MI	Cases	502	490	464	554	489		
	Model1	1.0 (ref.)	0.91 (0.80, 1.03)	0.82 (0.72, 0.93)	0.93 (0.82, 1.05)	0.79 (0.70, 0.89)	0.001	0.93 (0.88, 0.97)
	Model2	1.0 (ref.)	0.96 (0.85, 1.09)	0.89 (0.78, 1.01)	1.01 (0.89, 1.14)	0.84 (0.74, 0.96)	0.03	0.95 (0.91, 0.99)
	Model3	1.0 (ref.)	0.96 (0.84, 1.08)	0.88 (0.77, 1.00)	1.00 (0.88, 1.13)	0.83 (0.73, 0.95)	0.02	0.94 (0.90, 0.99)
	Model3	1.0 (ref.)	0.95 (0.84, 1.07)	0.86 (0.76, 0.98)	0.98 (0.87, 1.11)	0.82 (0.72, 0.93)	0.007	0.94 (0.89, 0.98)
Non-fatal AIS	Cases	348	342	326	316	380		
	Model1	1.0 (ref.)	0.91 (0.78, 1.06)	0.82 (0.71, 0.95)	0.75 (0.65, 0.88)	0.87 (0.75, 1.01)	0.02	0.94 (0.88, 0.99)
	Model2	1.0 (ref.)	0.99 (0.85, 1.15)	0.92 (0.79, 1.08)	0.87 (0.74, 1.01)	0.98 (0.85, 1.14)	0.49	0.98 (0.93, 1.04)
	Model3	1.0 (ref.)	0.99 (0.85, 1.15)	0.92 (0.79, 1.07)	0.86 (0.74, 1.01)	0.98 (0.85, 1.14)	0.48	0.98 (0.93, 1.04)
	Model3	1.0 (ref.)	0.97 (0.84, 1.13)	0.90 (0.77, 1.05)	0.84 (0.72, 0.98)	0.95 (0.82, 1.11)	0.26	0.97 (0.91, 1.02)
	**Ratio of Sodium to Potassium**							
Non-fatal MI	Cases	404	470	468	542	615		
	Model1	1.0 (ref.)	1.16 (1.01, 1.32)	1.15 (1.01, 1.32)	1.35 (1.18, 1.53)	1.55 (1.37, 1.76)	<0.0001	1.25 (1.18, 1.32)
	Model2	1.0 (ref.)	1.14 (1.00, 1.30)	1.09 (0.95, 1.25)	1.23 (1.08, 1.40)	1.32 (1.17, 1.50)	<0.0001	1.14 (1.08, 1.21)
	Model3	1.0 (ref.)	1.13 (0.99, 1.30)	1.08 (0.95, 1.24)	1.22 (1.07, 1.38)	1.31 (1.15, 1.49)	<0.0001	1.14 (1.07, 1.21)
	Model3	1.0 (ref.)	1.12 (0.98, 1.28)	1.06 (0.93, 1.22)	1.19 (1.04, 1.35)	1.27 (1.12, 1.45)	0.0001	1.12 (1.06, 1.19)
Non-Fatal AIS	Cases	312	330	314	317	439		
	Model1	1.0 (ref.)	1.06 (0.91, 1.24)	1.02 (0.87, 1.19)	1.05 (0.90, 1.23)	1.49 (1.29, 1.72)	<0.0001	1.22 (1.14, 1.31)
	Model2	1.0 (ref.)	1.08 (0.92, 1.26)	1.00 (0.86, 1.17)	0.99 (0.85, 1.16)	1.32 (1.14, 1.53)	0.0005	1.13 (1.06, 1.22)
	Model3	1.0 (ref.)	1.08 (0.92, 1.26)	1.00 (0.85, 1.17)	0.98 (0.84, 1.15)	1.31 (1.13, 1.52)	0.0006	1.13 (1.05, 1.21)
	Model4	1.0 (ref.)	1.06 (0.91, 1.24)	0.97 (0.83, 1.14)	0.95 (0.81, 1.11)	1.26 (1.08, 1.46)	0.005	1.11 (1.03, 1.19)

**Table 4:** Stratified analysis of the associations between dietary ratio of sodium to potassium and cardiovascular diseases in 180,156 participants from the Million Veteran Program (2011 to 2018).

	Quintiles of Dietary Indices					*p*_trend_	HR (95%CI)
	Q1	Q2	Q3	Q4	Q5		Per 1-SD Increment
**Sex**						*p* for interaction = 0.99	
Female	1.0 (ref.)	0.96 (0.64, 1.42)	0.66 (0.41, 1.04)	0.96 (0.62, 1.49)	1.36 (0.90, 2.06)	0.23	1.14 (0.92, 1.42)
Male	1.0 (ref.)	1.12 (1.00, 1.24)	1.04 (0.94, 1.16)	1.09 (0.98, 1.21)	1.26 (1.14, 1.40)	<0.0001	1.11 (1.06, 1.16)
**Race**						*p* for interaction = 0.92	
White	1.0 (ref.)	1.09 (0.98, 1.23)	1.03 (0.92, 1.15)	1.08 (0.97, 1.21)	1.28 (1.15, 1.43)	<0.0001	1.12 (1.07, 1.18)
Black	1.0 (ref.)	1.25 (0.95, 1.64)	0.96 (0.72, 1.28)	1.18 (0.90, 1.54)	1.29 (1.00, 1.66)	0.07	1.12 (0.99, 1.26)
**Baseline diabetes**						*p* for interaction = 0.99	
No	1.0 (ref.)	1.02 (0.89, 1.17)	0.94 (0.82, 1.08)	1.04 (0.91, 1.19)	1.21 (1.06, 1.38)	0.002	1.11 (1.04, 1.18)
Yes	1.0 (ref.)	1.20 (0.97, 1.48)	1.08 (0.88, 1.33)	1.15 (0.94, 1.40)	1.29 (1.06, 1.56)	0.02	1.11 (1.01, 1.21)
**Baseline high cholesterol**						*p* for interaction = 0.24	
No	1.0 (ref.)	1.17 (0.99, 1.38)	1.02 (0.85, 1.21)	1.06 (0.89, 1.26)	1.37 (1.17, 1.61)	0.0004	1.15 (1.06, 1.24)
Yes	1.0 (ref.)	1.00 (0.86, 1.16)	0.96 (0.82, 1.11)	1.07 (0.92, 1.24)	1.12 (0.97, 1.30)	0.04	1.07 (1.00, 1.15)
**Baseline hypertension**						*p* for interaction = 0.41	
No	1.0 (ref.)	0.99 (0.82, 1.20)	0.89 (0.73, 1.09)	0.96 (0.79, 1.16)	1.27 (1.06, 1.53)	0.008	1.13 (1.03, 1.24)
Yes	1.0 (ref.)	1.12 (0.97, 1.28)	1.03 (0.90, 1.19)	1.12 (0.98, 1.29)	1.22 (1.06, 1.39)	0.005	1.09 (1.03, 1.16)
**DASH score**						*p* for interaction = 0.07	
Low (≤21)	1.0 (ref.)	1.07 (0.87, 1.32)	0.99 (0.81, 1.21)	1.07 (0.88, 1.29)	1.25 (1.04, 1.49)	0.0003	1.14 (1.06, 1.22)
High (>21)	1.0 (ref.)	1.09 (0.97, 1.23)	1.00 (0.88, 1.13)	1.02 (0.89, 1.17)	1.10 (0.94, 1.28)	0.50	1.02 (0.95, 1.10)

Adjusted for age (continuous), sex (male or female, race/ethnicity (European Americans or other), education level (≤high school or GED, some colleague, or college or above), income level (<$30,000, $30,000–$59,000 or ≥$60,000) and marital status (currently married or not), smoking status (current, former or never smoking), frequency of alcohol consumption (never, <1 times/week or ≥1 times/week), frequency of exercise vigorously (never/rarely, 1–4 times/month, 2–4 times/week, or ≥5 times/week), total energy intake (in quintiles), and body mass index (continuous), presences of diabetes, high cholesterol, and hypertension at baseline (each Yes vs. No), except the stratified factor.

## Data Availability

Data will not be made available in order to comply with current VA privacy regulations.

## Supplementary Materials

The following supporting information can be downloaded at: https://www.mdpi.com/article/10.3390/nu14051121/s1, VA Million Veteran Program.
